# Improving the nutritional and sensory attributes of green faba bean seeds by whey-based fermentation

**DOI:** 10.1038/s41598-025-32435-7

**Published:** 2025-12-28

**Authors:** Heba Sayed Mostafa

**Affiliations:** 1 Food Science Department, Faculty of Agriculture, Cairo University, Giza, 12613 Egypt; 2 National Committee of Nutrition, Academy of Scientific Research and Technology, Cairo, Egypt

**Keywords:** Brine, Legumes, Plant-based protein, Vicia faba, Whey, Biotechnology, Microbiology

## Abstract

Fermentation enhances the nutritional and sensory properties of foods, including legumes like green faba beans. This study examines the effect of the fermentation medium (brine vs. whey) on trypsin inhibitor content and other physicochemical and sensory parameters of green faba bean seeds. Principal Component Analysis (PCA) was used to explore correlations between these features. Seeds were fermented for 7 days in either brine or whey, at 0%, 2%, 4%, 6%, or 8% salt. Data showed that lactic acid bacteria (LAB) growth was highest **(***p* < 0.05**)** in whey at low salt levels, accompanied by increased acidity. Trypsin inhibitor activity significantly decreased in both media, improving bean digestibility. The highest reduction (79.63%) occurred in whey at 2% salt. Brine-fermented beans exhibited lower lightness (L*), lower ash content, and a shift in a* values toward red compared to whey-fermented beans. Texture softened in both media, with whey producing a softer texture, particularly at higher salt levels. Based on PCA, the significant positive loadings of log LAB, acidity, pH, moisture, and L* on PC1 indicate that these variables are important drivers of change in the dataset, most likely reflecting the overall progress and features of the fermentation process. The distinct separation of fermentation methods in the PCA plot indicates that whey fermentation resulted in a more pronounced shift in bean attributes than brine fermentation, especially in terms of acidity and moisture content. Panelists preferred whey-fermented beans, particularly at 2% and 4% salt, for color and taste over brine-fermented and raw seeds. This study highlights whey fermentation’s potential to enhance green faba bean digestibility and nutritional profile, offering valuable implications for the food industry and consumer health.

## Introduction

Fermentation is a classic preservation process that improves the nutritional and sensory properties of food. Microorganisms break down complex organic substances, resulting in a variety of useful metabolic byproducts such as organic acids, vitamins, and bioactive chemicals^1,2^. Fermenting legumes and vegetables has received significant attention in recent years for its potential health advantages and its function in increasing food digestibility and nutritional quality^3^. During the microbial fermentation process, many metabolic activities and bio-transformation events occur, leading to a variety of health benefits, including anticancer^4^, anti-obesity^5^, anti-inflammatory^6^, and anti-aging^7^ effects. One of the primary advantages of legume fermentation is the elimination of antinutritional components such as trypsin inhibitors, which can impair protein digestion and absorption in the human body^8^. While several fermented products are derived from legumes, the majority are made from soybeans^9^. However, other legumes warrant further investigation.

Green faba bean, a popular legume recognized for its high nutrient content, is widely consumed in a variety of nations, including the Mediterranean, Egypt, China, India, Australia, Afghanistan, and northern Europe^10,11^. It can be consumed raw or cooked and used for a variety of purposes, including human food and cattle feed^12^. It contains trypsin inhibitors, which are proteins that inhibit the activity of trypsin, a digestive enzyme involved in protein breakdown^13^. Trypsin inhibitors are known to have certain health benefits, including possible anti-inflammatory and anti-cancer properties^14^, but when taken in excess, they can also cause problems with protein digestion^15^. Numerous processing techniques, including soaking (12.7% reduction), cooking presoaked beans (100.0% reduction)^16^, microwaving (42.0% reduction), and autoclaving (84.0% reduction)^17^, have been used to reduce these inhibitors in faba beans. Fermentation has been shown to reduce the levels of these inhibitors, enhancing the digestibility and nutritional value of the beans^18–20^. Many bacterial strains have shown the ability to reduce trypsin inhibitors through the production of acid proteases^21^. When 13 varieties of lactic acid-fermented faba bean flour were analyzed, fermentation demonstrated a promising trypsin inhibitor reduction, ranging from 66.67 to 89.65%^22^. Coda^23^ assessed the effects of lactic acid fermentation on tannins, phytates, and protease inhibitors in faba bean flour. The highest documented reductions were 72% for trypsin inhibitors, 45% for phytates, and 50% for tannins. Faba bean flour was also fermented with lyophilized yogurt cultures, which resulted in a modest 4.7% reduction in trypsin inhibitor activity^24^.

The use of brine and whey as fermentation media provides an interesting contrast. Brine solution is often used in food preservation and fermentation, such as olives, because it promotes the growth of beneficial microbes like lactic acid bacteria (LAB) while limiting the growth of rotting organisms^25,26^. Whey, on the other hand, is a byproduct of dairy production that comprises roughly 93% water, 4.8% lactose, 0.8% protein, 0.5% fat, and minerals^27^. Its composition has been demonstrated to promote the growth of lactic acid bacteria and other microorganisms that contribute to fermentation^28^. Fermentation has been used to valorize whey for the production of prebiotics, peptides, and exopolysaccharides^29^. It has also been utilized to supplement fermented dairy products^30^, alcoholic and non-alcoholic beverages^31,32^, and fermented meat products^33^. However, the extent to which fermentation of whole legumes in different media, such as whey, remains an area of ongoing research.

The research hypothesizes that whey, due to its richer nutrient composition, would enhance lactic acid bacteria growth and fermentation efficiency compared to the traditional brine medium. By comparing both media, this study specifically aims to evaluate how different fermentation media (brine vs. whey) influence the nutritional, physicochemical, and sensory attributes of green faba bean seeds, with a particular focus on reducing trypsin inhibitor activity to enhance protein digestibility. The findings are intended to provide a practical approach for utilizing whey, a dairy by-product, as a cost-effective fermentation medium for producing value-added legume-based foods in the food industry.

## Materials and methods

### Plant materials and chemicals

Fresh green faba bean (*Vicia faba* L.) pods were purchased from a local market, peeled, and the collected beans were washed to remove any dirt or foreign particles. Pasteurized whey, obtained as a byproduct of mozzarella cheese manufacture (acidity 0.42%, pH 6.0, lactose content 4.5%), was acquired from the Dairy Department, Faculty of Agriculture, Cairo University. Casein, trypsin from porcine pancreas, Bradford reagent, and MRS (de Man, Rogosa, and Sharpe) agar medium were purchased from Sigma-Aldrich, St. Louis, MO, USA. All other reagents were of analytical grade.

### Sample preparation

To prepare the fermented samples, 100 g of freshly collected green faba beans were placed in sterile 250-mL glass jars with two different fermentation media (150 mL). The glass jars were thoroughly washed and then sterilized in a hot air oven at 160 °C for one hour to ensure a sterile starting environment. Distilled water or whey with varying salt concentrations (0, 2, 4, 6, and 8%) was used as the submerged fermentation medium. Each tested medium was poured over the beans, and acetic acid (1%, v/v) was added and mixed. The fermentation jars were kept sealed to prevent contamination from external microorganisms. The fermentation took place under controlled conditions (25 °C ± 2) for a total of 7 days in the absence of light. After the fermentation period, fermented samples were analyzed for changes in trypsin inhibitor content, microbial content, and sensory properties and compared with the fresh, unfermented samples.

### Microbial examination

After the 7-day fermentation period, the LAB count in the fermentation medium was determined as log CFU/mL using the pour-plate count method. One mL of the fermentation solution was serially diluted in sterile saline (0.85%), and 1.0 mL of each dilution was plated before pouring MRS agar medium and anaerobically incubated at 37 °C for 72 h^34^.

### Physicochemical examination

To evaluate moisture content, green faba beans were dried to a constant weight at 105 °C for approximately 3 h. The ash content was determined by combusting the green faba beans at 550 °C in a muffle furnace till a constant weight was achieved. The pH of the fermentation medium was determined using a digital pH meter (Adwa AD1030 pH meter, Romania) calibrated with standard buffer solutions (pH 4.0 and 9.0). Total titratable acidity was measured as lactic acid by titrating a 10 mL sample of the fermentation solution with NaOH (0.1 N) until it turned pink and expressed as a percentage of lactic acid (w/v). The changes in acidity were examined to determine the course of fermentation^11^.

Color analysis of green faba beans before and after fermentation was performed in triplicate using a Minolta chromameter (CR-410, Tokyo, Japan), and L*, a*, b*, Chroma, and Hue were recorded. The texture of the green beans was assessed with a fruit pressure tester (FT 02 Penetrometer, Norfolk, VA 23502, USA), which measures the force necessary to compress the beans, and the force (in Newton) was measured as an indication of firmness.

### Trypsin inhibitor activity examination

The activity of trypsin inhibitors was evaluated using a modified Zhang technique^35^. The experiment involves homogenizing the bean sample (10 g) in distilled water (10 mL) to extract the proteins, including trypsin inhibitors, and centrifuging for 15 min at 2795 × g by Hermle, Z300 (Germany). The extract (0.2 mL) was mixed with 1.6 mL of casein solution (1% in 0.1 M Tris-HCl, pH 7.5) and 0.2 mL of trypsin (50 mg/100 mL). The test tubes were incubated for 15 min at 37 °C. The reaction was terminated by adding 0.4 mL of trichloroacetic acid solution (5% w/v). The absorbance of the diluted mixture and trypsin standard, which represents the casein concentration after reaction, was measured spectrophotometrically at 280 nm (Unico-UV2000, USA). Trypsin inhibitory activity is defined as U/mg of the sample and calculated by this equation.

$${\text{Trypsin inhibitor activity (U/mg dw)}} = \frac{Astd-Asample}{Sample\:weight\:}\times {\text{Dilution factor}}$$

where A_std_ = Absorbance of the standard and A_sample_ = Absorbance of the sample.

### Protein digestibility examination

It was tested as described by Verni^22^. First, 0.5 g of each homogenized bean sample was added to 20 mL HCl (0.1 M) with 1.5 mg/mL pepsin and incubated for 3 h at 37 °C. Subsequently, 10 mL of NaOH (0.5 M) + 10 mL phosphate buffer (0.2 M, pH 8.0) containing 10 mg trypsin were added, and the tubes were incubated at 37 °C for 24 h. To stop the reaction, one mL of 10% TCA (w/v) was added and centrifuged at 2795 × g for 15 min. Protein concentration in the supernatant was spectrophotometrically measured before and after digestion with Bradford reagent at A595, and digestibility was calculated as the percentage of the total protein after enzyme digestion using this equation.

$${\text{Protein digestibility}} (\%) =\:\frac{(Non\:digested\:sample-Digested\:sample)}{Nondigested\:sample} \times 100$$

### Sensory examination

A consumer-type sensory evaluation was conducted using 40 untrained panelists (25 women and 15 men, aged 18–30) from the Food Science Department, Faculty of Agriculture, Cairo University. All individuals gave informed consent, and the formal approval was waived by Al-Azhar University ethics committee (ID: AZHAR 5/25), as Cairo University does not have its own Research Ethics Committee for sensory evaluation. All tests were performed in accordance with the relevant guidelines and regulations of the Institute of Food Science & Technology (IFST)^36^. At room temperature (25 °C), freshly fermented samples were placed in coded white plates with a slice of bread, presented in a randomized order to minimize positional and expectation bias, and rated using 9-hedonic scales. A scale of 1 represents “dislike,” and a scale of 9 represents “like extremely.” The panelists were asked to evaluate 9 samples for the following attributes: appearance (color), taste (sourness, saltiness, or overall flavor), odor, texture (firmness/crunchiness), aroma, and overall acceptability^37^. Blinding was maintained by ensuring panelists were unaware of the fermentation medium or salt concentration during evaluation.

### Statistical analysis

All experiments were carried out in triplicate, and the results were evaluated using analysis of variance (ANOVA) to see how the fermentation medium (brine solution vs. whey) and salt content affected the various parameters. Tukey’s HSD test was used to compare means, with a significance level of *p* < 0.05^38^. Principal Component Analysis (PCA) was performed to investigate the correlations between the physicochemical features of fermented beans and to reduce data dimensionality while preserving the most significant variation. PCA was performed with the free PAST software (version 4.03; Natural History Museum, University of Oslo, Norway)^39^. The PCA was conducted using a correlation matrix, which better compensates for discrepancies in measurement scales and highlights correlations between variables. The resulting biplot shows the samples plotted based on their PC1 and PC2 scores, with loading vectors reflecting the influence of each original variable on the principal components.

## Results and discussion

### Microbial count

The results in Table 1 illustrate how the fermentation medium and its salt content affect microbial growth (Log CFU/mL), acidity, and pH. LAB counts decreased noticeably as salt increased, indicating the inhibitory effect of high salinity. At all equivalent salt levels, whey supported significantly higher LAB growth than brine (*p* < 0.05), reflecting its richer nutrient content. The data for the 0% salt whey treatment represent a key finding, demonstrating that salt remains essential for controlled fermentation. This confirms that even in a nutrient-rich medium like whey, salt is crucial for steering fermentation toward a stable and desirable product.

**Table 1:** Lactic acid bacteria count, acidity, and pH of the fermentation media after fermentation of green faba bean seeds for 7 days.

Fermentation medium	Salt conc. %	LAB (Log CFU/mL)	Acidity % w/v	pH
Fresh	–	–	0.09^j^ ± 0.003	6.95^a^ ± 0.00
Brine	2	3.93^dB^ ± 0.005	0.30^fB^ ± 0.002	4.32^eA^ ± 0.02
	4	3.87^eB^ ± 0.016	0.27^gB^ ± 0.001	4.47^eA^ ± 0.04
	6	2.96^fB^ ± 0.013	0.22^hB^ ± 0.002	4.74^dA^ ± 0.04
	8	2.93^fB^ ± 0.000	0.19^iB^ ± 0.002	4.99^cA^ ± 0.04
Whey	0	2.89^f^ 0.002 ±	0.42^eA^ ± 0.008	5.55^bB^ ± 0.02
	2	7.62^aA^ ± 0.007	1.22^aA^ ± 0.000	3.34^hB^ ± 0.05
	4	7.59^aA^ ± 0.007	1.20^bA^ ± 0.002	3.54^gB^ ± 0.05
	6	7.43^bA^ ± 0.004	1.03^cA^ ± 0.008	3.63^gB^ ± 0.03
	8	6.39^cA^ ± 0.003	0.99^dA^ ± 0.007	3.96^fB^ ± 0.05

*All values were expressed as the mean ± SD of three determinations. Different small letters within the same column indicate statistically significant differences between the samples. Capital letters indicate statistically significant differences between the brine and whey samples at the same salt concentration (*p*-value < 0.05).

The highest LAB count occurred at 2% salt in whey (7.62 ± 0.007 log CFU/mL), decreasing progressively at 6% and 8% due to osmotic stress (6.39 ± 0.003 log CFU/mL at 8% salt). Whey’s composition, including its lactose (4.8%), proteins (0.8%), and bioavailable minerals and vitamins^27^, likely promoted greater microbial proliferation compared to brine, which lacks fermentable substrates. Lactose can be biologically transformed into various high-value products, including lactic acid, a primary metabolite in LAB fermentation^40^. Whey also contains up to 90% of the initial calcium concentration found in milk, which is highly bioavailable. This mineral is essential for various metabolic activities in LAB, including enzyme activation and stabilization^39^. Moderate salt levels enhanced microbial performance, whereas excessive salinity reduced microbial activity, consistent with previous reports^41^. This study quantified only total LAB counts without identifying specific strains. Future work employing 16 S rRNA sequencing or selective plating is needed to determine the dominant microbial species and their specific roles in fermentation. Comparable LAB counts (3.40 and 6.00 log CFU/g) have been reported during natural fermentation of bean flour (*Phaseolus vulgaris* L.) after 8 and 24 h, respectively, supporting the patterns observed in this study.

Acidity reflected microbial activity during fermentation and varied with both medium and salt level. In brine, acidity decreased significantly from 0.30 ± 0.002 at 2% salt to 0.19 ± 0.002 at 8%, indicating reduced acid production as salt increasingly inhibited microbial growth. Whey showed significantly higher acidity than brine at all corresponding salt levels (*p* < 0.05), although acidity also declined with increasing salt concentration for the same reason. The whey used was sweet mozzarella whey, which was acidified with 1% acetic acid to promote LAB dominance and suppress spoilage microbes. Thus, the higher acidity in whey resulted from both the added acid and subsequent microbial fermentation. Whey provides a more supportive environment for acid production due to its lactose and organic acid content, as well as the potential for lactic acid to thrive and greater acid accumulation during fermentation^40,44^, which can enhance preservation, color development, and flavor. As expected, pH values showed the opposite pattern, corresponding with observed LAB counts. These trends agree with previous findings, such as Liang^45^, who reported decreasing acidity and lactic acid levels at higher salt concentrations (9%) in fermented cabbage (*Brassica rapa pekinensis*) compared to 2% and 4% salt levels.

### Physicochemical attributes

Table 2 illustrates the physicochemical attributes (moisture, ash, and texture) of the fermented faba bean seeds. Fermentation significantly increased the moisture content of the faba beans compared to the fresh sample (70.61% ± 1.74 dw), which is a characteristic effect of liquid-based fermentation systems. There were no significant differences (*p* ≥ 0.05) in moisture content between brine and whey fermentation at corresponding salt levels. The 0% salt whey sample had a moisture content of 80.15% ± 0.02 dw, significantly higher than the fresh beans, indicating that water absorption occurred even in the absence of salt. In both media, the moisture content of the seeds was high at 2% salt and did not significantly decrease as the salt concentration increased. As the salt concentration rises, water is likely pulled out of the beans by osmosis, resulting in a slight steady decline in moisture content. Obviously, fermentation is a more effective approach to retaining moisture in fresh green beans than other traditional preservation procedures such as drying, which may affect moisture (reaching 7.67–11.33%), texture, and overall quality^46^.

**Table 2:** Physicochemical attributes of green faba bean seeds fermented in two fermentation media at varying salt levels.

Fermentation medium	Salt conc. %	Moisture (% dry weight)	Ash (% dry weight)	Texture (*N*)
Fresh	–	70.61^bB^ ± 1.74	1.17^cA^ ± 0.31	2.10^aA^ ± 0.36
Brine	2	86.05^aA^ ± 3.12	2.29^bcA^ ± 0.65	0.07^eB^ ± 0.02
	4	82.92^aA^ ± 1.81	2.57^bcB^ ± 0.10	0.12^deB^ ± 0.02
	6	80.40^abA^ ± 6.41	3.43^abB^ ± 0.20	0.58^bcA^ ± 0.02
	8	79.64^abA^ ± 2.30	3.60^abB^ ± 0.54	0.83^bA^ ± 0.05
Whey	0	80.15^abA^ ± 0.02	1.33^cA^ ± 0.15	0.22^cdeB^ ± 0.17
	2	84.12^aA^ ± 0.86	2.44^bcA^ ± 0.40	0.27^cdeA^ ± 0.02
	4	81.39^abA^ ± 3.02	3.51^abA^ ± 0.06	0.40^cdeA^ ± 0.05
	6	77.41^abA^ ± 2.40	4.13^aA^ ± 0.10	0.42^cdeB^ ± 0.07
	8	74.04^abA^ ± 3.76	4.25^aA^ ± 0.50	0.48^bcB^ ± 0.02

*All values were expressed as the mean ± SD of three determinations. Different small letters within the same column indicate statistically significant differences between the samples. Capital letters indicate statistically significant differences between the brine and whey samples at the same salt concentration (*p*-value < 0.05).

The ash content of the faba beans increased with fermentation, which is consistent with the absorption of minerals from the fermentation media. The fresh sample had the lowest ash content as 1.17% ± 0.31 dw. In both brine and whey, ash content increased with rising salt concentration and significantly differed at 6% and 8% salt compared to low salt levels. A key finding was the statistically significant difference in ash content between the two media, with whey-fermented samples consistently containing a higher percentage of ash than their brine-fermented counterparts at every tested salt concentration (*p* < 0.05), especially at concentrations ≥ 4%. This is most likely due to the naturally higher mineral content of whey, which contains Ca^2+^, Mg^2+^, and K^+^^47^, facilitating mineral absorption by the beans during fermentation. This enrichment may enhance nutritional value and position fermented whey-beans as a nutrient-dense food option.

The texture of green faba bean seeds was significantly altered by fermentation in both brine and whey media. As shown in Table 2, fermentation in both media led to a substantial reduction in texture, with the lowest value recorded in brine with 2% salt (0.07 ± 0.02 N). The effect of salt concentration was also pronounced, with texture values significantly increased with a higher salt percentage in brine fermentation. This can be accredited to the osmotic pressure exerted by the salt, which can inhibit the activity of the microbial enzymes (pectinases and cellulases) or suppress LAB growth, leading to reduced cell wall degradation. A statistically significant difference (*p* < 0.05) in texture was observed between the two media at all salt concentrations, with the brine-fermented samples being softer at 2% and 4% salt, while the whey-fermented samples were softer at 6% and 8% salt. This softening is likely due to hydrolytic enzyme activity, particularly pectinases and cellulases produced by LAB or other microbes during fermentation, which break down the structural polysaccharides in the bean cell wall^48^. In comparison, the hardness of green bell pepper increased after lactic acid fermentation^49^, whereas the hardness of Xiaomila pepper (*Capsicum frutescens* L.) varieties decreased significantly after fermentation, which is consistent with our experiment^50^.

### Color

The color attributes of the green faba bean seeds were significantly affected by the fermentation process, with notable differences observed between the brine and whey fermentation media. The color measurements, represented by the L*, a*, b*, Chroma, and Hue values, provide a quantitative assessment of these changes (Table 3). The L* value indicates lightness, where higher values correspond to a brighter color. The a* value represents the green-to-red axis, with negative values indicating a more green hue. Fresh green faba bean seeds exhibited a high L* value (62.50 ± 0.05) and a significantly negative a* value (-7.82 ± 0.03), confirming their bright green appearance. Upon fermentation in brine, a general darkening of the seeds was observed, as reflected by a decrease in L* values across all fermented samples compared to the fresh control. In contrast, the L* values of the whey-fermented seeds were generally higher than the brine-fermented seeds, indicating better lightness retention at comparable salt concentrations.

**Table 3:** Color attribute scores of green faba bean seeds fermented in two fermentation media at varying salt levels.

Fermentation medium	Salt conc. %	L*	a*	b*	Chroma	Hue
Fresh	-	62.50^bA^ ± 0.05	-7.82^hA^ ± 0.03	17.07^aB^ ± 0.07	18.78^aA^ ± 0.07	114.62^aA^ ± 0.02
Brine	2	57.47^dB^ ± 0.07	-0.20^cA^ ± 0.02	7.07^eB^ ± 0.12	7.07^fB^ ± 0.12	97.13^dA^ ± 0.08
	4	55.50^eB^ ± 0.05	-0.16^cA^ ± 0.02	6.99^efB^ ± 0.07	6.99^fB^ ± 0.07	97.32^dA^ ± 0.08
	6	55.01^eB^ ± 0.33	0.17^bA^ ± 0.00	6.73^fB^ ± 0.09	6.73^fB^ ± 0.09	101.44^cA^ ± 0.13
	8	53.23^fB^ ± 0.00	0.58^aA^ ± 0.01	4.70^gB^ ± 0.08	4.79^gB^ ± 0.04	101.17^cA^ ± 0.04
Whey	0	63.2^abA^ ± 1.01	-6.76^gB^ ± 0.15	17.23^aA^ ± 0.06	18.51^bB^ ± 0.10	111.24^bB^ ± 0.43
	2	64.04^aA^ ± 0.03	-1.58^dB^ ± 0.02	11.73^dA^ ± 0.16	13.89^cA^ ± 0.09	83.11^gB^ ± 0.37
	4	63.30^abA^ ± 0.14	-1.61^dB^ ± 0.02	12.62^cA^ ± 0.07	12.72^dA^ ± 0.08	88.54^fB^ ± 0.09
	6	63.08^abA^ ± 0.03	-2.32^eB^ ± 0.03	12.52^cA^ ± 0.02	12.62^dA^ ± 0.02	91.46^eB^ ± 0.32
	8	60.30^cA^ ± 0.13	-2.75^fB^ ± 0.04	13.62^bA^ ± 0.08	11.92^eA^ ± 0.12	91.34^eB^ ± 0.15

*All values were expressed as the mean ± SD of three determinations. Different small letters within the same column indicate statistically significant differences between the samples. Capital letters indicate statistically significant differences between the brine and whey samples at the same salt concentration (*p*-value < 0.05).

A key finding was the difference in color preservation between the two fermentation media. Faba bean seeds fermented in whey consistently retained a greener color compared to those fermented in brine. This is evident from the significantly lower (more negative) a* values in the whey samples. For instance, at 4% salt concentration, the a* value for the brine sample was − 0.16 ± 0.02, while the whey sample’s a* was − 1.61 ± 0.02, indicating a greater retention of green pigment. The a* value in brine samples increased with rising salt content, moving toward a less green tint, while the opposite was true for whey-fermented samples, suggesting enhanced stability of green pigments. Additionally, whey-fermented samples showed higher b* values than brine-fermented samples at the same salt level, suggesting a more intense and yellow-tinted color.

The values of a* and b* influenced the Chroma (intensity) and Hue (vibrancy) values of faba green beans. By comparing both media, whey-fermented samples had significantly higher Chroma values (ranging from 13.89 ± 0.09 at 2% to 11.92 ± 0.12 at 8%) than samples fermented in brine solution at the same salt level. Additionally, Hue values significantly (*p* < 0.05) increased with increasing salt concentrations. These changes are likely attributed to the combined effects of fermentation on chlorophyll stability and cellular structure. Fermentation, an acidic process, often leads to the degradation of chlorophyll into pheophytin, a brown-green pigment, resulting in the loss of the characteristic bright green color^51^. The shift in the a* value from a strong negative to a value closer to zero (or even positive) in the brine samples is a direct indicator of this chlorophyll-to-pheophytin conversion. The superior color preservation in the whey medium is likely due to its unique chemical composition and the microbial community it supports. LAB present in whey samples (Table 1) produces lactic acid, which may contribute to slower chlorophyll degradation. One possible explanation is that the resulting acidic conditions may inhibit the activity of chlorophyllase^52^, the enzyme responsible for chlorophyll breakdown; however, this mechanism remains hypothetical in the absence of direct enzymatic measurements. This observation was also detected in lactic acid fermented *Solanum scabrum* and *Solanum villosum* vegetables^53^, and *Brassica campestris* L. leaves^54^.

### Nutritional attributes

**Fig. 1:**
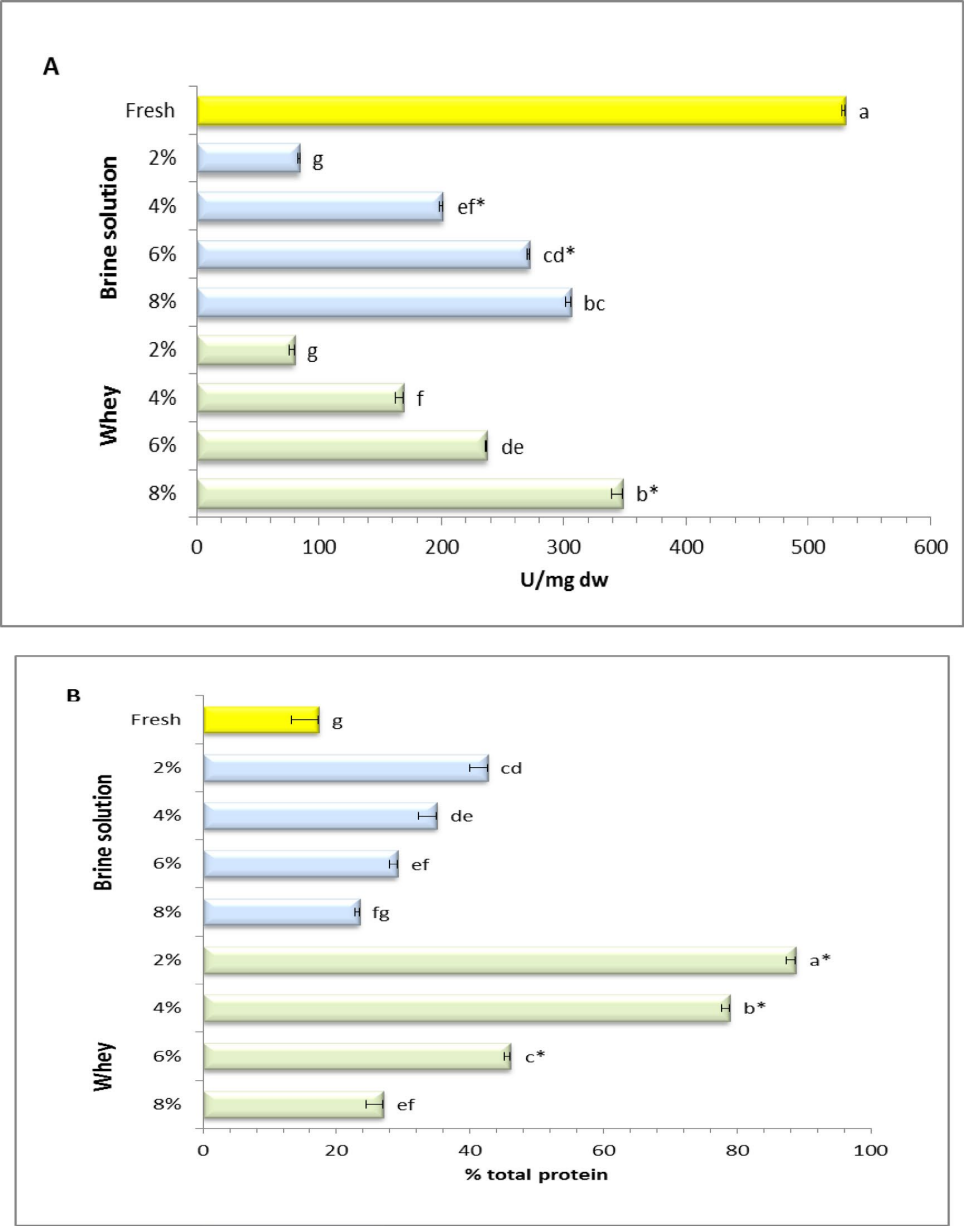
Trypsin inhibitor activity (**A**) and protein digestibility (**B**) of fermented faba bean seeds in two fermentation media (brine vs. whey) at varying salt levels. *Indicate significant difference between brine and whey at the same salt level. †Different small letters indicate statistically significant differences between the samples (*p*-value < 0.05).

The results (Fig. 1A) show that trypsin inhibitor content in green beans decreased significantly after fermentation in both brine and whey media. Fresh beans recorded the highest value (530.11 ± 23.76 U/mg), while the greatest reduction occurred at 2% salt in whey medium (79.63% ± 4.12). Trypsin inhibitor levels increased gradually with higher salt concentrations but remained below those of the fresh sample. Overall, whey-fermented samples exhibited lower inhibitor levels than brine, except at 8% salt, indicating a more effective reduction, likely due to LAB activity and protein-modifying conditions associated with whey. This may be attributed to the presence of bioactive compounds in whey that can stimulate LAB growth and activity. Whey protein contains a variety of bioactive peptides, such as lactoferrin, α-lactalbumin, and β-lactoglobulin, which can modulate the growth and activity of LAB^55^. LAB present in the fermenting medium produce a range of extracellular and intracellular proteases. These enzymes are highly effective at hydrolyzing proteins, including the protein-based trypsin inhibitors, rendering them inactive and thus reducing their anti-nutritional effects^56^. The LAB-induced increase in acidity further lowers the pH, creating conditions that can also promote irreversible denaturation of trypsin inhibitor proteins. As their three-dimensional structure is essential for their function, this denaturation effectively inactivates them, resulting in a reduction in their activity^57^.

Protein digestibility (Fig. 1B) showed an opposite pattern and aligned with previous findings in legumes, where processing and fermentation substantially reduce trypsin inhibitors. For example, soaking, microwaving, and autoclaving resulted in reductions of 12.7%, 42.0%, and 84.0%, respectively^16,17^, while fermentation of faba bean flour by *Lactobacillus plantarum* DPPMAB24W resulted in a reduction ranging from 66.67% to 89.65%^22^, which aligns well with the current study.

Although these findings demonstrate the positive nutritional effects of fermentation, the overall nutritional implications cannot be fully assessed because this study did not measure parameters such as amino acid profile or mineral content. This represents a limitation of the current work, and future studies should include detailed compositional analyses to better determine the nutritional significance of whey-based fermentation.

### Principal component analysis

**Fig. 2:**
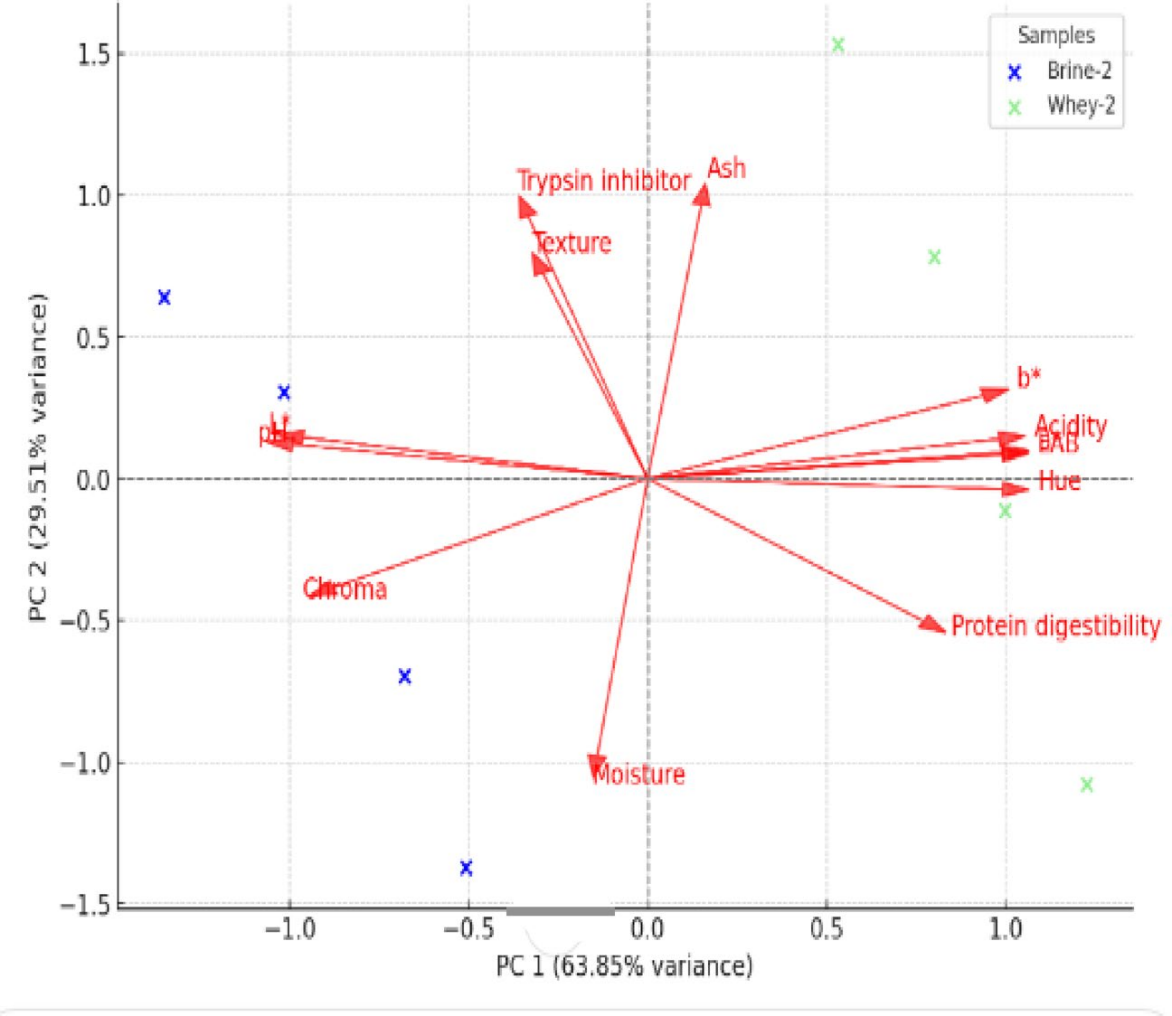
Principal component analysis (PCA) based on the physicochemical and nutritional features of faba bean seeds fermented in brine or whey at varying salt levels.

PCA was conducted to reduce the dimensionality of the dataset while retaining the most essential changes among the variables. The PCA model includes the following variables: log LAB, acidity, pH, moisture, ash, texture, trypsin inhibitor, protein digestibility, L*, a*, b*, Chroma, and Hue. The first two principal components (PC1 and PC2) explained 63.85% and 29.51% of the overall variance, respectively, for a total of 93.36% of the variation in the dataset (Fig. 2). Given the substantial cumulative variance, PC1 and PC2 were deemed sufficient to depict the primary trends in the data. The PCA biplot shows distinct clustering patterns based on fermentation type (brine vs. whey). Brine-fermented bean samples (blue dots) cluster along the negative PC1 axis, whereas whey-fermented faba bean samples (light green points) are more evenly distributed, notably along the positive PC1 axis. This distribution suggests that whey and brine fermentation exert different influences on the physicochemical characteristics of the beans. The significant positive loadings of log LAB, acidity, pH, moisture, and L* on PC1 indicate that these variables are important drivers of change in the dataset, most likely reflecting the overall progress and features of the fermentation process. PC2 can be understood as representing textural properties. The high loading of texture on PC2 demonstrates its importance in identifying samples along this dimension, which could be due to changes in fermentation conditions or microbial activity that affect texture. The distinct separation of fermentation methods in the PCA plot indicates that whey fermentation causes a higher shift in bean attributes than brine fermentation, notably in terms of acidity and moisture content. The findings emphasize the possibility of modifying fermentation methods to attain desired bean qualities based on specific processing objectives.

### Sensory attributes

Table 4 shows that fermentation noticeably changed the sensory properties of faba beans and that the fermentation medium had a stronger influence than salt concentration. Whey-fermented samples consistently scored higher than brine-fermented ones across all attributes, with the exception of overall acceptability at 8% salt, where both were similar. While brine samples generally scored lower than fresh beans, whey-fermented beans maintained high taste and overall acceptability scores; for instance, the 4% whey sample (8.1 ± 1.10) exceeded the fresh sample (7.4 ± 0.52). This improvement is likely due to enhanced LAB activity in whey, resulting in balanced production of organic acids and flavor compounds.

**Table 4:** Sensory evaluation scores of green faba bean seeds fermented in two fermentation media at varying salt levels.

Fermentation medium	Salt conc. %	Color	Taste	Odor	Texture	Over all acceptability
Fresh	-	8.7^aA^± 0.48	7.4^abA^ ± 0.52	8.7^aA^ ± 0.67	8.2^aA^ ± 1.03	8.0^abcA^ ± 0.82
Brine	2	4.4^fB^ ± 0.52	5.6^cB^ ± 0.70	4.2^fB^ ± 0.79	5.5^cB^ ± 1.08	5.5^eB^ ± 0.71
	4	5.2^efB^ ± 0.63	5.3^cdB^ ± 0.67	4.7^fB^ ± 1.25	6.1^cB^ ± 0.99	6.0^deB^ ± 0.82
	6	5.8^deB^ ± 0.63	5.0^cdB^ ± 0.67	5.0^efB^ ± 0.67	6.6^bcB^ ± 1.17	5.8^cdB^ ± 1.03
	8	6.2^dB^ ± 0.42	4.6^dB^ ± 0.52	5.2^efB^ ± 0.63	6.9^abcB^ ± 0.52	7.2^bcdA^ ± 0.42
	0	8.5^aA^ ± 0.52	7.6^abA^ ± 0.51	8.4^abA^ ± 0.69	7.8^abA^ ± 1.03	7.8^abcA^ ± 0.63
Whey	2	7.4^bcA^ ± 0.70	7.4^abA^ ± 0.70	7.5^bcA^ ± 0.85	7.6^abcA^ ± 0.52	8.5^aA^ ± 0.97
	4	8.3^abA^ ± 1.06	8.1^aA^ ± 1.10	7.1^cdA^ ± 1.20	7.8^abA^ ± 1.32	8.3^abA^ ± 1.06
	6	8.0^abcA^ ± 0.67	7.9^abA^ ± 0.99	6.6^cdA^ ± 0.70	8.0^abA^ ± 0.94	7.8^abcA^ ± 0.92
	8	7.3^cA^ ± 0.48	7.2^bA^ ± 0.63	6.0^deA^ ± 0.63	8.1^aA^ ± 0.88	7.5^abcA^ ± 0.71

*All values were expressed as the mean ± SD of three determinations. Different small letters within the same column indicate statistically significant differences between the samples. Capital letters indicate statistically significant differences between the brine and whey samples at the same salt concentration (*p*-value < 0.05).

Salt concentration had different effects in each medium. In whey, 0% salt produced sensory scores similar to the fresh beans, indicating minimal preference for fermentation at that level. Increasing salt improved the sensory quality of brine samples (*p* < 0.05), but in whey it did not consistently enhance attributes except texture. The best taste score occurred at 4% salt, while the highest overall acceptability (8.5 ± 0.97) was observed at 2% in whey medium, supporting the potential for developing lower-salt fermented products.

The desirable flavors and textures are associated with high LAB activity, which generates key metabolites during the anaerobic fermentation (e.g., ethyl acetate, ethyl propanoate, diacetyl, acetoin, and esters)^58–60^ that shape the aroma and taste of fermented foods. This may explain why certain fermented samples received lower odor scores than the raw beans. During the digestion of food items, these bacteria also produce lactic, acetic, and propionic acids, which give the product a sour flavor. Organic acids, alcohol, and aldehydes combine to produce a variety of flavor chemicals that improve the taste of the fermented product^9^. Future studies should characterize these metabolites in the most preferred fermentation conditions.

## Conclusion

This study successfully demonstrates, for the first time, the feasibility and benefits of using whey as a sustainable fermentation medium for green faba bean seeds. Our findings show that the choice of fermentation medium (whey vs. brine) and salt concentration are critical factors that significantly influence the fermentation process and the final product quality. Whey, due to its richer nutritional composition, supported more robust microbial growth than brine, resulting in a faster and more efficient fermentation. A moderate salt concentration (2–4%) proved to be optimal, effectively promoting microbial activity and ensuring a successful fermentation without the inhibitory effects observed at higher concentrations (6–8%). Beyond the fermentation kinetics, whey-fermented faba beans exhibited superior quality attributes, including a more vibrant green color and higher sensory acceptability. PCA supported these outcomes, revealing strong positive correlations between LAB growth and desirable qualities such as acidity, moisture, and color (L* value). In conclusion, this study introduces a promising approach to valorizing dairy by-products while producing a nutritious, value-added food product from faba beans. However, the work has certain limitations. Future research should focus on the metagenomic analysis to identify the specific microbial species responsible for the fermentation in each medium, as well as a detailed nutritional assessment (e.g., amino acid and vitamin profiles) of the final product to fully characterize its functional properties.

## Data Availability

The data that support the findings of this study are available from the corresponding author, H.M., upon reasonable request.
